# Pyruvate Kinase M2 Nuclear Translocation Regulate Ferroptosis-Associated Acute Lung Injury in Cytokine Storm

**DOI:** 10.1007/s10753-024-02000-x

**Published:** 2024-03-14

**Authors:** Haiting Wang, Chenyu Fan, Xuelian Chen, Wei Zhou, Li Guo, Feng Zhao, Shuang Ye, Shuangjun He, Yi Chen

**Affiliations:** 1 Department of Rheumatology, Renji Hospital, Shanghai Jiao Tong University School of Medicine, Shanghai, China; 2 Department of Emergency and Critical Care Medicine, Renji Hospital, Shanghai Jiao Tong University School of Medicine, Shanghai, China; 3 Department of Burn, Ruijin Hospital, Shanghai Jiao Tong University School of Medicine, Shanghai, China; 4 Department of Immunology and Microbiology, Shanghai Institute of Immunology, Shanghai Jiao Tong University School of Medicine, Shanghai, China

**Keywords:** cytokine storm, acute lung injury, ferroptosis, pyruvate kinase M2

## Abstract

Cytokine storm (CS) is linked with macrophage dysfunction and acute lung injury (ALI), which can lead to patient mortality. Glycolysis is preferentially exploited by the pro-inflammatory macrophages, in which pyruvate kinase M2 (PKM2) is a critical enzyme. The mechanism underlying the link between CS and ALI involves cell death, with the recently discovered programmed cell death known as ferroptosis being involved. However, the relationship between the glycolysis and ferroptosis in the context of CS-related ALI remains unclear. CS-associated ALI induced by poly I:C (10 mg/kg, i.v) and LPS (5 mg/kg, i.p) (IC: LPS) exhibit significant ferroptosis. Ferrostatin-1 (ferroptosis inhibitor) treatment attenuated IC:LPS‑induced mortality and lung injury. Moreover, Alveolar macrophage (AM) from IC:LPS model exhibited enhanced glycolysis and PKM2 translocation. The administration of ML-265(PKM2 monomer/dimer inhibitor) resulted in the formation of a highly active tetrameric PKM2, leading to improved survival and attenuation of ALI. Furthermore, ML-265 treatment decreased ferroptosis and restored the balance between anaerobic glycolysis and oxidative phosphorylation. Notably, in patients with lung infection, intracellular expression level of PKM2 were correlated with circulating inflammation. Enhanced ferroptosis and PKM2 nuclear translocation was noticed in CD14^+^ blood monocytes of lung infection patients with CS. In conclusion, PKM2 is a key regulatory node integrating metabolic reprograming with intra-nuclear function for the regulation of ferroptosis. Targeting PKM2 could be explored as a potential means in the future to prevent or alleviate hyper-inflammatory state or cytokines storm syndrome with aberrant ferroptotic cell death.

**Supplementary Information:**

The online version contains supplementary material available at 10.1007/s10753-024-02000-x.

## Introduction

The cytokine storm (CS), a hyperactivated immune response, is one of the largest current issues worldwide since the advent of the COVID-19 pandemic [1, 2]. It has been linked with acute lung injury (ALI) and multiple organ failure [3]. Innate immune cells are central to the CS pathogenesis because they express a range of pattern recognition receptors, including Toll-like receptors (TLRs), which are activated by endogenous and exogenous inflammatory signals [4]. Murine models of CS have demonstrated that sustained TLR activation is sufficient to amplify proinflammatory cytokine production and induce CS-mediated immunopathology [5]. Similarly, successive Poly I:C (TLR3) and LPS (TLR4) (IC: LPS) activation in wild type mice may induce clinical manifestations of CS, including hepatosplenomegaly, hepatitis, cytopenia, and hypercytokinemia [6]. Patients with CS syndrome exhibit increased numbers of pulmonary inflammatory macrophages, which are also linked to macrophage dysfunction [7], suggesting that macrophages may be a therapeutic target for inhibiting the CS syndrome. Elevated levels of several inflammatory factors are typical features of a CS, which can lead to an unfavorable prognosis [8, 9]. However, blocking pro-inflammatory cytokines such as TNF-a, IL-1, or IL-6 have had inconsistent results in the treatment of the CS syndrome [4, 10], suggesting a lack of a consistent pathogenic process. Thus, a novel targeted macrophage therapeutic paradigm, which interferes with the signaling of pro-inflammatory cytokines, is required.

Ferroptosis is a newly discovered form of programmed cell death associated with iron-mediated lipid free radical accumulation and dysregulation of antioxidant responses [11]. Ferroptosis is involved in the pathophysiological progression of cancer [12], cardiovascular diseases [13], and infections [14]. LPS and Poly I:C are sufficient to cause severe oxidative stress, with excessive generation of reactive oxygen species (ROS) [15] and an increase in ferrous iron (Fe2^+^) in the labile iron pool [16], which can catalyze the production of ROS via Fenton chemistry, leading to oxidative stress. Corroborating these experimental findings, patients with CS present with elevated serum ferritin, a heteropolymeric protein complex composed of 24 ferritin heavy/ferritin light (FTH/FTL) subunits that can store and convert Fe2^+^ into inert Fe3^+^ through the ferroxidase activity of FTH to counteract the disrupted cellular iron homeostasis [17]. The role of ferroptosis in CS is attracting increasing attention. However, previous studies have mostly focused on the key pathways of ferroptosis, including mitochondrial ROS, GPX4, and GSH/GSSH. Concurrently, the sensitivity of ferroptosis seems to be highly dependent on the state of cellular lipid [18] and amino acids [19] metabolism. Some studies have suggested that the inhibition of hexokinase (HK; an enzyme in anaerobic glycolytic metabolism) by 2-deoxy-d-glucose (2-DG)[20] or glucose starvation could substantially inhibit lipid peroxidation and ferroptosis [21]. However, the exact mechanism of glucose metabolism involved in ferroptosis during CS remains elusive.

Conventionally, glycolysis is preferentially exploited by pro-inflammatory macrophages, in which pyruvate kinase M2 (PKM2) is a critical enzyme. Previously, Wang *et al*. showed that tumor cells undergoing ferroptosis have substantially lower PKM2 enzyme activity [22]. PKM2 is known to exist in two conformations: a tetramer, which is cytosol-localized and functions as enzymatically active as a pyruvate kinase, and a dimer form, which is enzymatically less active than the tetrameric isoform [23] and can translocate into the nucleus, wherein it can interact with HIF1α to regulate glycolytic gene expression [24]. These conformations may be interconverted in response to specific stimuli or injuries. Furthermore, PKM2 tetramerization may reduce ROS levels [25] and correct the pro-inflammatory phenotype of macrophages in coronary artery disease [26]. However, whether conformational changes in PKM2 can regulate ferroptosis-associated ALI remains unknown.

Herein, we aimed to demonstrate the underlying mechanism by which ML-265, a small-molecule activator, affects CS-related ALI by inhibiting ferroptosis through the induction of tetrameric PKM2. Our results demonstrate the remarkable plasticity of glucose metabolism in macrophages, suggesting new ways of facilitating the resolution of ferroptosis in CS-related ALI.

## Results

### Cytokines Storm Associated Acute Lung Injury Induced By Poly I:C and LPS Exhibit Significant Ferroptosis

**Fig. 1:**
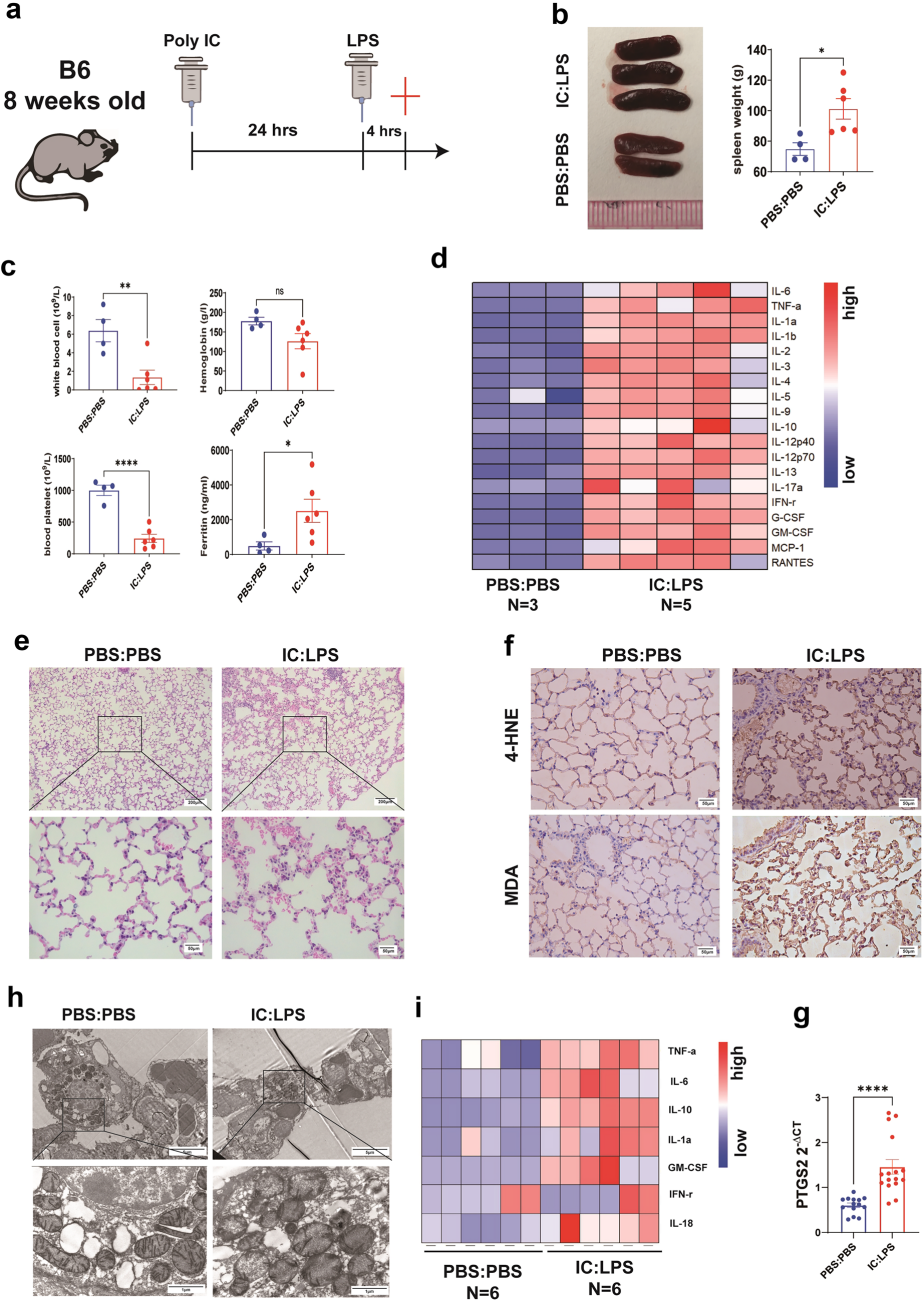
Cytokine storm associated with acute lung injury induced by Poly I:C and LPS exhibits significant ferroptosis (**a**) Experimental protocol. LPS(5 mg/kg) was injected intraperitoneally at 24 h after poly I:C (10 mg/kg) injection. Mice were scarified 4 h after the second challenge. Splenomegaly (**b**), blood routine test (**c**), plasma ferritin (**c**) and plasma cytokines (**d**) were measured. H&E staining (**e**), immunohistochemical staining of 4-HNE and MDA (**f**) in lung sections were measured. G. qRT-PCR analysis of PTGS2 in lung cells. Gene expression was normalized to Gapdh. H. Mitochondrial morphology was measured in lung sections using transmission electron microscopy. I. Heatmap of qRT-PCR analysis of cytokine gene expression in lung cells. Gene expression was normalized to Gapdh. Data are represented as mean ± SD. P < 0.05 was considered significant. *P < 0.05, **P < 0.01, ****P < 0.0001.

Compared with the most common LPS-induced lung injury model, there has been no research on lung injury and ferroptosis involvement in a mouse model sequentially challenged with poly I:C and LPS (Fig. 1a). Consistent with a previous report [6], the IC:LPS model showed splenomegaly (Fig. 1b), leukopenia (Fig. 1c), thrombocytopenia (Fig. 1c), and dramatically elevated levels of ferritin (Fig. 1c) and serum pro-inflammatory cytokines (Fig. 1d). This model exhibited features of ALI, including widened alveolar septa, fluid accumulation within the alveolar space, and inflammatory cell infiltration (Fig. 1e). Moreover, the IC:LPS model displayed notably higher levels of 4-hydroxynonenal (4-HNE) and malondialdehyde (MDA) compared to that displayed by the control mice (Fig. 1f). In addition, the mRNA expression of prostaglandin-endoperoxide synthase-2 (Ptgs2), another typical feature of ferroptosis [27], was elevated in the IC:LPS model (Fig. 1g). Concurrently, we examined changes in mitochondrial morphology, a crucial event in ferroptosis execution [28]. Electron microscopy of lung cells revealed shrunken mitochondria, disruption of the inner cristae, and rupture of the outer membrane (Fig. 1h). Heightened ferroptosis rate was accompanied by an increased level of *in situ* expression of inflammatory cytokines in the lungs (Fig. 1i).

### Fer-1 Treatment Attenuated IC:LPS‑Induced Lung Injury in Mice

**Fig. 2:**
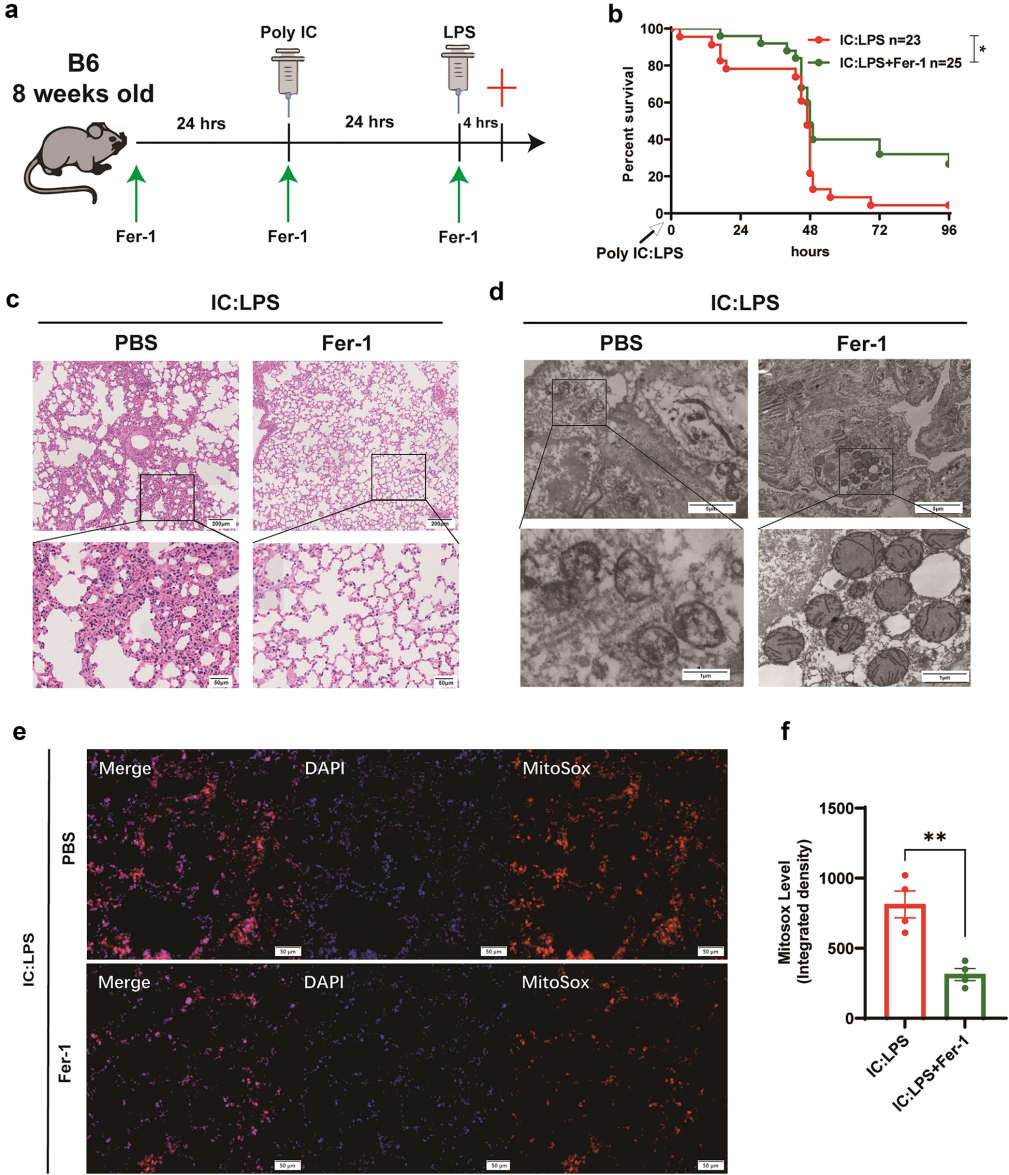
Fer-1 treatment attenuated IC:LPS‑induced lung injury in mice (**a**) Experimental protocol. LPS(5 mg/kg) was injected intraperitoneally at 24 h after poly I:C (10 mg/kg) injection. Where indicated, the mice were administered a daily intraperitoneally of Fer-1(2.5 mg/kg) for three consecutive days, starting one day before poly I:C treatment. Mice were scarified 4 h after the second challenge. (**b**) Survival of animals challenged with IC:LPS between two groups. H&E staining (**c**) and mitochondrial morphology (**d**) in lung sections were measured. Confocal imaging (**e**) and quantification (**f**) of mitosox (red) labeling in the lung. Data are represented as mean ± SD. P < 0.05 was considered significant. *P < 0.05, **P < 0.01.

To investigate the relative contribution of ferroptosis in IC:LPS-induced CS model related-ALI, we measured systemic inflammatory responses and survival in mice in the absence or presence of ferroptosis inhibitor (ferrostatin-1, a synthetic antioxidant; Fer-1 [29]). Mice underwent a 3-day Fer-1 treatment starting a day before poly: IC administration (Fig. 2a). In comparison to the high mortality rate observed within 96 h after IC:LPS challenge, the survival rate increased to 30% in Fer-1 treated mice (p = 0.021) (Fig. 2b). Concurrently, Fer-1 mitigated the histopathological lesions, which manifested as reduced inflammatory cell infiltration and diminished alveolar collapse (Fig. 2c). The Fer-1 treatment group exhibited enhanced mitochondrial morphology (Fig. 2d), along with a notable reduction in mitochondrial ROS levels, as measured using MitoSox Red (Fig. 2e, f). To further verify the role of ferroptosis in IC:LPS‑induced lung injury, we used another specific and mechanistically distinct ferroptosis inhibitor (deferasirox; an oral iron chelator) in our mouse model (Supplemental Fig. 1a) and similarly found that deferasirox treatment could reduce mortality by 20% (Supplemental Fig. 1b) while alleviating histopathological lung injury and mitochondrial damage (Supplemental Fig. 1c, d). Our results revealed that the IC:LPS-induced CS resulted in significant lung injury and increased ferroptosis. However, this change was reversed by the ferroptosis inhibitor treatment.

### Alveolar Macrophage (AM) Exhibit Enhanced Glycolysis and PKM2 Translocation in IC:LPS-Induced ALI

**Fig. 3:**
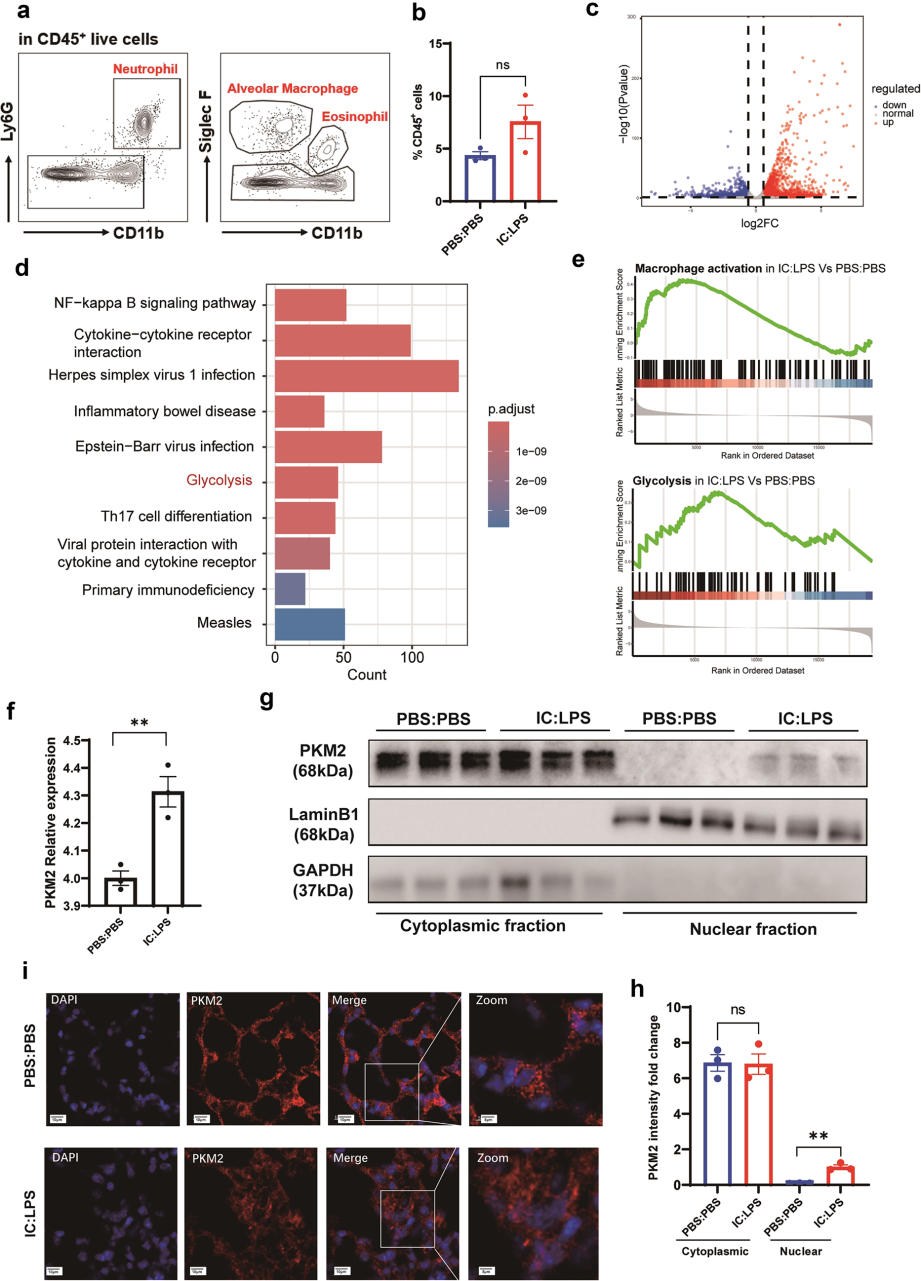
Alveolar macrophages exhibit enhanced glycolysis and PKM2 translocation in IC:LPS-induced ALI (**a**) Gating strategy for the sorting of AM (CD45^+^ Ly6G^−^ Siglec F^+^ CD11b^int/lo^) for RNA-seq. (**b**) Percentage of AM in CD45 + cells in lung. (**c**)Volcano plot showing DEGs of AM between IC:LPS treated and PBS:PBS treated groups. (**d**) Pathway analysis between IC:LPS treated and PBS:PBS treated groups. (**e**) Upregulation of inflammatory and glycolysis pathways of AM in the IC:LPS group compared with PBS:PBS treated group. (**f**) Expression of PKM2 in AM. (G-H) Western blot analysis of PKM2 expression in cytoplasmic and nuclear fraction of AM. (I) Confocal imaging of PKM2 (red) and DAPI (blue) labeling in the lung. Data are represented as mean ± SD. P < 0.05 was considered significant. **P < 0.01.

Activated immune cells, such as macrophages, possess the ability to shift their energy metabolism from oxidative phosphorylation to glycolysis [6]. This metabolic switch directly influences the regulation of the inflammatory response. However, the relationship between inflammatory response, glycolysis, and ferroptosis remains unclear. To discern transcriptional variations, we conducted bulk RNA-seq on AM obtained from IC:LPS-induced model and control group (Fig. 3a). While the percentage of AM in the IC:LPS group exhibited an increasing trend in lung infiltration compared to that in the PBS group, the difference was not statistically significant (Fig. 3b). We identified differentially expressed AM genes between the two groups and focused our analyses on differentially activated pathways (Fig. 3c, d). AM in the IC:LPS group exhibited upregulation of inflammatory pathways accompanied by an elevation in glycolysis (Fig. 3d, e). In contrast, AM downregulated the inflammatory pathways in mice treated with IC: LPS plus Fer-1 (data not shown).

Since the metabolic analyses of AM revealed a preference for glycolysis, we further examined the metabolic enzymes involved in this pathway. PKM2, which governs the last step of glycolysis, is a rate-limiting enzyme. There are two isoforms of pyruvate kinase M (PKM1 and PKM2) [30]. Recent studies have revealed that PKM2 contributes to the activation of macrophages and release of inflammatory factors [31]. Growing evidence suggests that the transition from PKM2 tetramer to PKM2 monomer/dimer (nuclear translocation) plays a crucial role in promoting inflammatory responses [32]. In our comparison of the two isoforms, we observed a significantly higher expression level of PKM2 in the IC:LPS group compared with that in the control group (Fig. 3f). To validate the RNA-seq results of RNA sequencing, western blotting and immunofluorescence staining were performed to measure the expression pattern of PKM2 in AM. Subcellular fractionation showed that nuclear PKM2 expression increased in the IC:LPS group, whereas the cytoplasmic fraction remained unchanged (Fig. 3g, h). Immunofluorescence confirmed the nuclear localization of PKM2 (Fig. 3i). These findings imply that the pathological progression of IC:LPS-induced ALI may be linked to increased translocation of PKM2, triggering an inflammatory response.

### Inhibition of PKM2 Nuclear Translocation Alleviates Lung Injury and Ferroptosis in IC:LPS Model

**Fig. 4:**
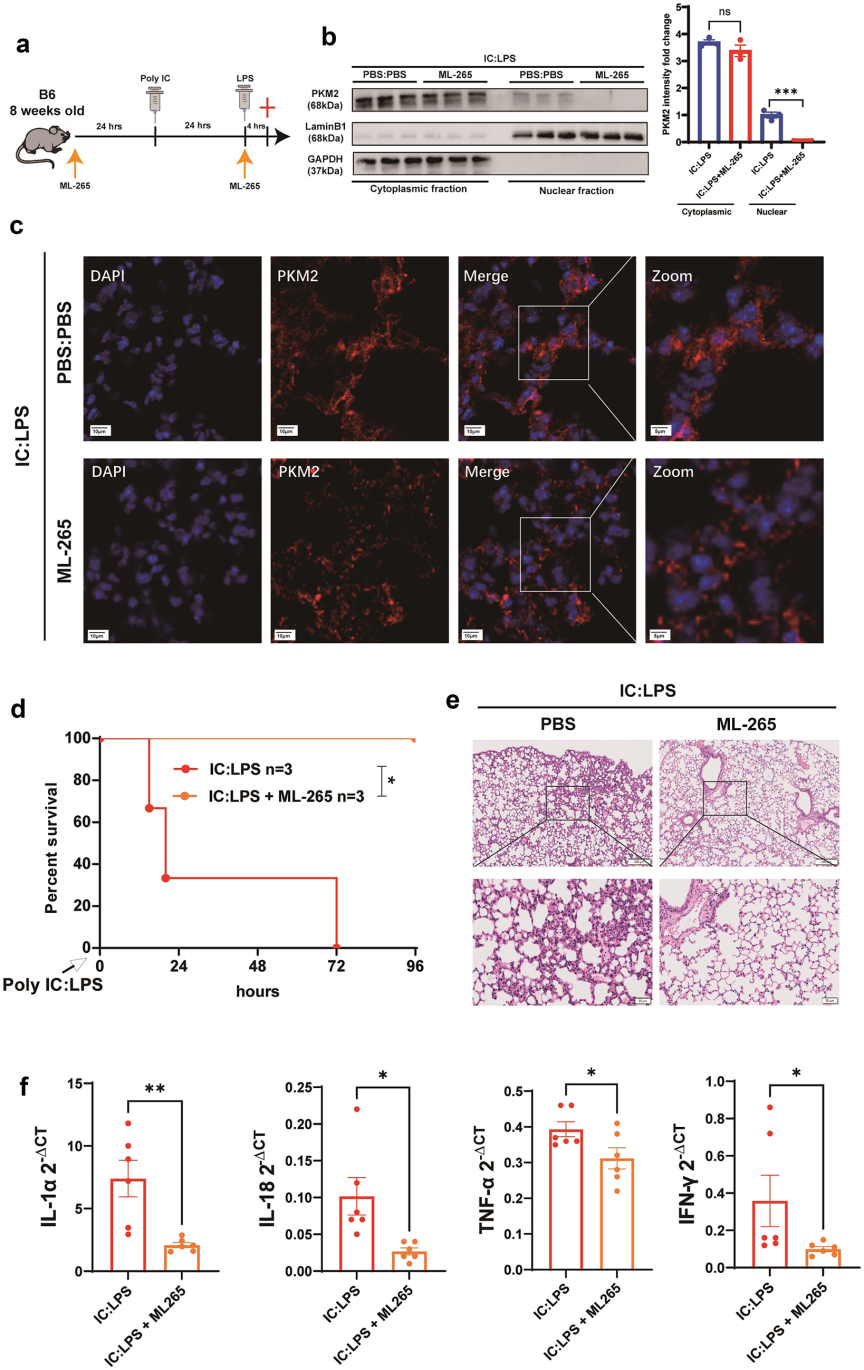
Inhibition of PKM2 nuclear translocation alleviates lung injury in IC:LPS model (**a**) Experimental protocol. LPS(5 mg/kg) was injected intraperitoneally at 24 h after poly I:C (10 mg/kg) injection. Where indicated, the mice were administered every other day intraperitoneally of ML-265 (50 mg/kg), starting one day before poly I:C treatment. Mice were scarified 4 h after the second challenge. (**b**-**c**) Western blot analysis of PKM2 expression in cytoplasmic and nuclear fraction of AM. (**c**) Confocal imaging of PKM2 (red) and DAPI (blue) labeling in the lung. (**d**) Survival of animals challenged with IC:LPS between two groups. (**e**) H&E staining in lung sections were measured. (**f**) qRT-PCR analysis of cytokine gene expression in lung cells. Gene expression was normalized to Gapdh. Data are represented as mean ± SD. P < 0.05 was considered significant. *P < 0.05, **P < 0.01, ***P < 0.001.

To further determine the effect of PKM2 nuclear translocation on lung injury in the IC:LPS model, mice were treated with IC:LPS with or without ML-265 (a PKM2 monomer/dimer inhibitor) [23] (Fig. 4a). The treatment of ML-265 abolished the nuclear localization of PKM2 in AM, as confirmed by immunoblotting (Fig. 4b) and confocal microscopy (Fig. 4c). Mice treated with ML-265 were resistant to mortality induced by IC:LPS, similar to the previously observed dramatic improvement in survival with 2-DG [6], indicating that PKM2-mediated glycolysis contributes to the fatal outcome associated with the CS (Fig. 4d). The survival change in the ML-265 group was accompanied by improved pathological changes (Fig. 4e) and decreased levels of inflammatory cytokines in the lungs (Fig. 4f).

**Fig. 5:**
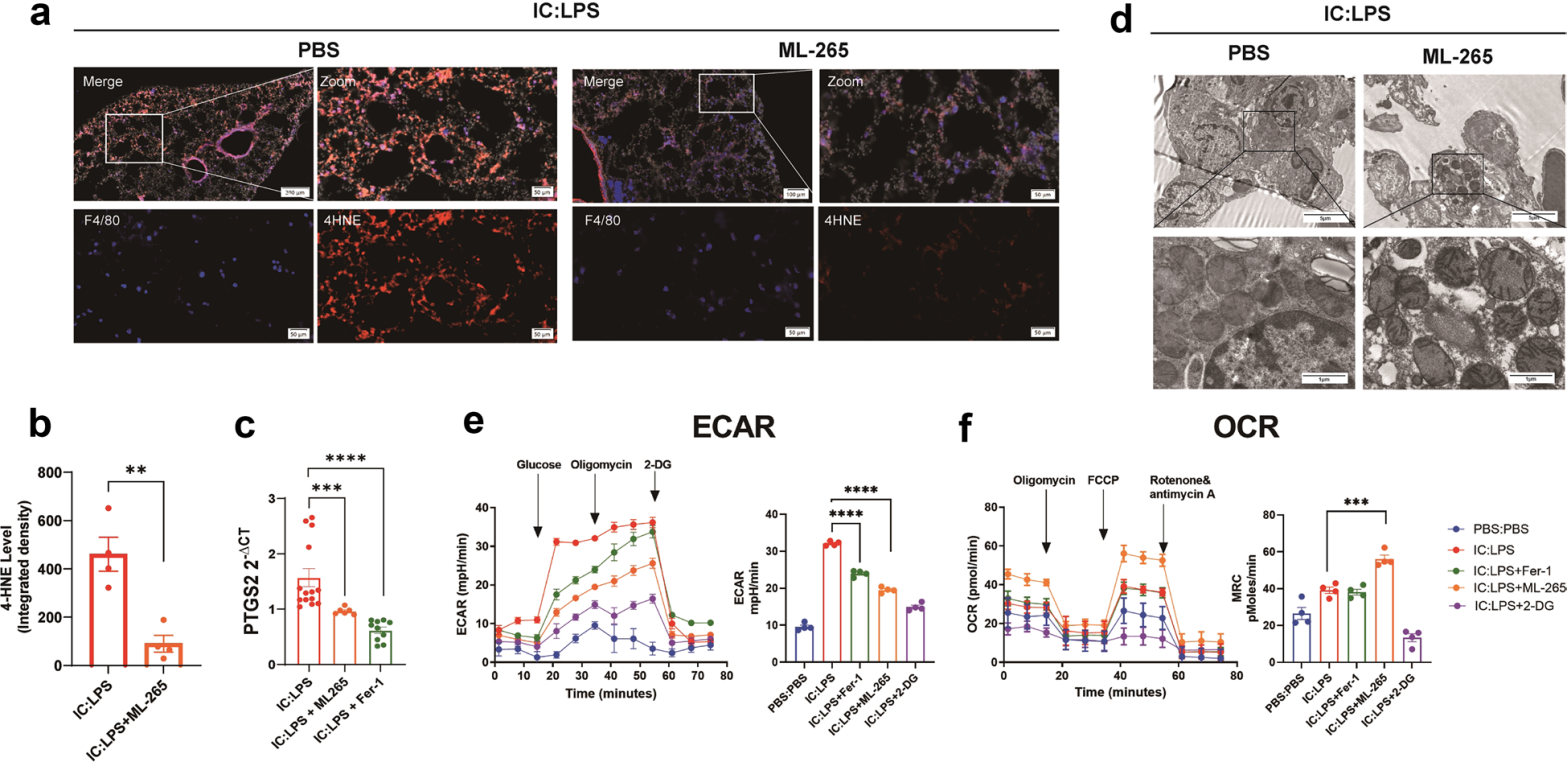
Inhibition of PKM2 nuclear translocation alleviates ferroptosis in IC:LPS model (**a**-**b**) Confocal imaging of 4-HNE (red) and F4/80 (blue) labeling in the lung. (**c**) qRT-PCR analysis of PTGS2 in lung cells. Gene expression was normalized to Gapdh. (**d**) Mitochondrial morphology in lung sections were measured. (**e**–**f**) The ECAR and OCR were measured in lung cells of mice in different groups by using a Seahorse XF96e Extracellular Flux analyzer. Data are represented as mean ± SD. P < 0.05 was considered significant. **P < 0.01, ***P < 0.001, ****P < 0.0001.

Next, we investigated whether the inhibition of PKM2 nuclear translocation could rescue ferroptosis. Mice pretreated with ML-265 exhibited a noteworthy decrease in the expression of 4-HNE compared to those treated with PBS (Fig. 5a, b). In addition, the ML-265 group exhibited a downregulation in the mRNA level of Ptgs2 (Fig. 5c) and an improvement in mitochondrial morphology in the lungs (Fig. 5c, d). The effect of ML-265 on the metabolic flux in lung cells was further assessed using seahorse assays. The evaluation of glycolysis through the ECAR revealed that both Fer-1 and ML-265 significantly reduced the glycolytic capacity (Fig. 5e). In contrast, the OCR, as measured by the maximal respiration capacity (MRC), was not significantly different between the IC: LPS and IC: LPS + Fer-1 control groups. However, ML-265 treatment resulted in a significant increase of MRC compared to IC: LPS group (Fig. 5e).

These data suggest that, in the IC:LPS-induced CS model, the administration of ML-265 resulted in the formation of a highly active tetrameric PKM2, leading to improved survival and attenuation of ALI. Furthermore, ML-265 treatment decreased ferroptosis and restored the balance between anaerobic glycolysis and oxidative phosphorylation.

### PKM2 Nuclear Translocation Associated with Circulating Inflammation in Patients with Lung Infection

**Fig. 6:**
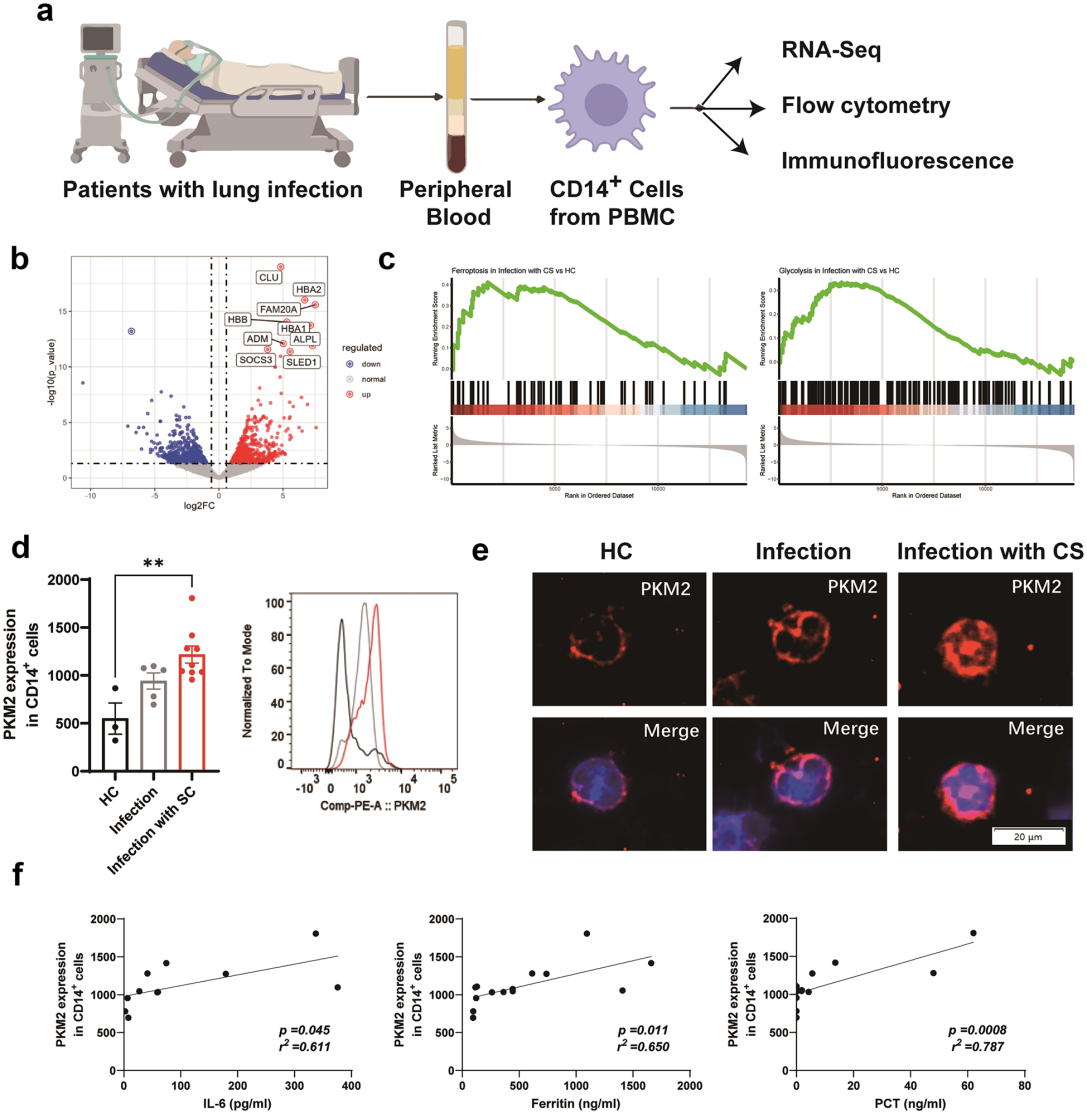
PKM2 nuclear translocation associated with circulating inflammation in patients with lung infection (**a**) Experimental protocol. Peripheral blood of 14 patients diagnosed with lung infection and 3 healthy controls (HC) were collected. CD14^+^ cells were isolated from PBMCs and further analyzed using RNA-Seq, flow cytometry, and immunofluorescence. (**b**) Volcano plot showing DEGs of CD14^+^ cells between lung infection patients with CS and healthy controls. (**c**) Upregulation of ferroptosis and glycolysis pathways of CD14^+^ cells in lung infection patients with CS compared with healthy controls. (**d**) Flow analysis (MFI value) of PKM2 expression in CD14^+^ cells. (**e**) Confocal imaging of PKM2 (red) and DAPI (blue) labeling in CD14^+^ cells. (**f**) Correlation of PKM2 expression in CD14^+^ cells with inflammatory parameters (IL-6, ferritin and PCT). Data are represented as mean ± SD. P < 0.05 was considered significant. **P < 0.01.

Classical CD14^+^ blood monocytes migrate into the lung tissue and give rise to human AM during inflammation [33]. Therefore, to further evaluate PKM2 nuclear translocation in CS-related ALI in humans, we performed a detailed multiparametric study combining transcriptomic analyses, flow cytometry, and immunofluorescence in CD14^+^ cells from PBMCs (Fig. 6a). Our study consisted of a cohort of 14 patients diagnosed with lung infection based on HRCT scans, with 9 of these patients exhibiting distinct characteristics suggestive of CS [34] (Table 1). Among the 9 patients, seven were confirmed to have SARS-CoV-2 infection, while two were confirmed to have cytomegalovirus infection. Additionally, we included a control group consisting of three healthy controls.

**Table 1:** Baseline characteristics of healthy controls, lung infection patients and lung infection with cytokine storm

**Variable**^b^	**Healthy controls (*n* = 3)**	**Lung infection (*n* = 5)**	**Lung infection with cytokine storm (n = 9)**	***P*** value*
Male, n (%)	3 (100.0)	3 (60.0)	9 (100.0)	0.999
Age (year), mean (SD)	38.0 (10.8)	70.8 (7.8)	72.7 (12.3)	< 0.001
Evidence of pathogens, n (%)^a^				
Bacterial	0 (0.0)	4 (80.0)	8 (88.9)	
Viral	0 (0.0)	1 (20.0)	9 (100)	
Fungal	0 (0.0)	0 (0.0)	1 (11.1)	
**Entry Criteria**				
Positive for COVID-19, n (%)	0 (0.0)	1 (20.0)	7 (77.8)	< 0.001
Ferritin (ug/L), median (IQR)	20 (2, 45)	115 (95, 1408)	443 (121, 1660)	< 0.001
C-reactive protein (mg/L), median (IQR)	5 (1, 8)	85 (9, 135)	120 (59, 205)	< 0.001
**Optional Criteria**				
Albumin (g/L), median (IQR)	45.0 (42.5–48.5)	28.6 (27.4–31.5)	22.7 (18.6–29.6)	< 0.001
Lymphocytes (10^9/L), median (IQR)	2.44 (2.44–4.33)	1.5 (1.5–2.1)	0.8 (0.6–0.9)	< 0.001
Neutrophil (10^9/L), median (IQR)	1.3 (1.3–1.7)	5.4 (4.3–12.2)	12.4 (9.7–18.7)	< 0.001
AST (mg/L), median (IQR)	13.00 (12.00–16.00)	28.0 (21.0–29.0)	62.0 (39.0–72.0)	0.024
D-dimers > 600 (mg/L), n (%)	0 (0.0)	1 (20.0)	5 (55.5)	0.414
LDH (mg/L), median (IQR)	85.0 (77.5–87.5)	217.0 (160.0–298.0)	348.0 (278.0–374.0)	0.017
Troponin I (ng/L), median (IQR)	0.01 (0.01–0.01)	0.01 (0.01–0.2)	1.0 (0.3–1.5)	0.007
BUN: creatinine ratio, median (IQR)	14 (13–15)	19 (17–23)	25 (21, 28)	0.024
Procalcitonin (ng/L), median (IQR)	0.03 (0.03–0.05)	4.27 (0.02–13.60)	4.27 (0.02–13.60)	0.012
ESR (mm/h), median (IQR)	8.0 (8.0–9.0)	27.0 (23.0–60.0)	35.0 (32.0–45.0)	< 0.001
IL-6 (pg/L), median (IQR)	2.3 (2.0–2.9)	13.6 (7.8–22.3)	60.0 (41.4–74.5)	< 0.001

HRCT, high-resolution computed tomography; AST, Glutamic oxalic aminotransferase; ESR, erythrocyte sedimentation rate; LDH, lactate dehydrogenase; BUN, Blood Urea Nitrogen

^*^P-values were calculated to compare the differences between the Healthy and Infection with cytokine storm groups. Comparison of continuous variables using student-t test and Mann–Whitney nonparametric test. Categorical variables were compared using chi-squared test or Fisher exact test

^a^Twelve cases were identified as bacterial infections, including 4 with Streptococcus pneumonia, 3 with Enterococcus faecium, and 5 with Klebsiella pneumonia. Viral infections totaled 10 cases, with 8 attributed to the COVID-19 and 2 to cytomegalovirus. One case was a fungal infection, caused by Cryptococcus neoformans

^b^Data are presented as median (interquartile range) or mean (standard deviation)

We isolated CD14^+^ cells from PBMCs (n = 3 in the healthy controls and n = 3 in CS infection groups) and generated RNA-seq data. We identified differentially expressed genes between the two groups (Fig. 6b). CD14^+^ blood monocytes in patients with CS exhibited an upregulation of ferroptosis accompanied by an increase in glycolysis (Fig. 6c), which is consistent with the results of our *in vivo* models. Intracellular expression of PKM2 in peripheral blood CD14^+^ monocytes was significantly higher in patients presenting with CS than in healthy controls (Fig. 6d). Immunofluorescence further confirmed that elevated intracellular PKM2 levels in these patients were accompanied by an increase in nuclear translocation (Fig. 6e). To determine whether PKM2 nuclear translocation can also occur in other white blood cells—T cells, B cells, and neutrophils, we performed immunofluorescence analysis of peripheral blood samples and did not find significant PKM2 nuclear translocation regardless of the patients of lung infection with or without CS (Supplemental Fig. 2). Furthermore, Correlation analysis also suggested a positive association between PKM2 expression in peripheral blood CD14^+^ monocytes and inflammatory markers, including serum IL-6, ferritin, and PCT (Fig. 6f), in patients with acute respiratory infection.

## Discussion

**Fig. 7:**
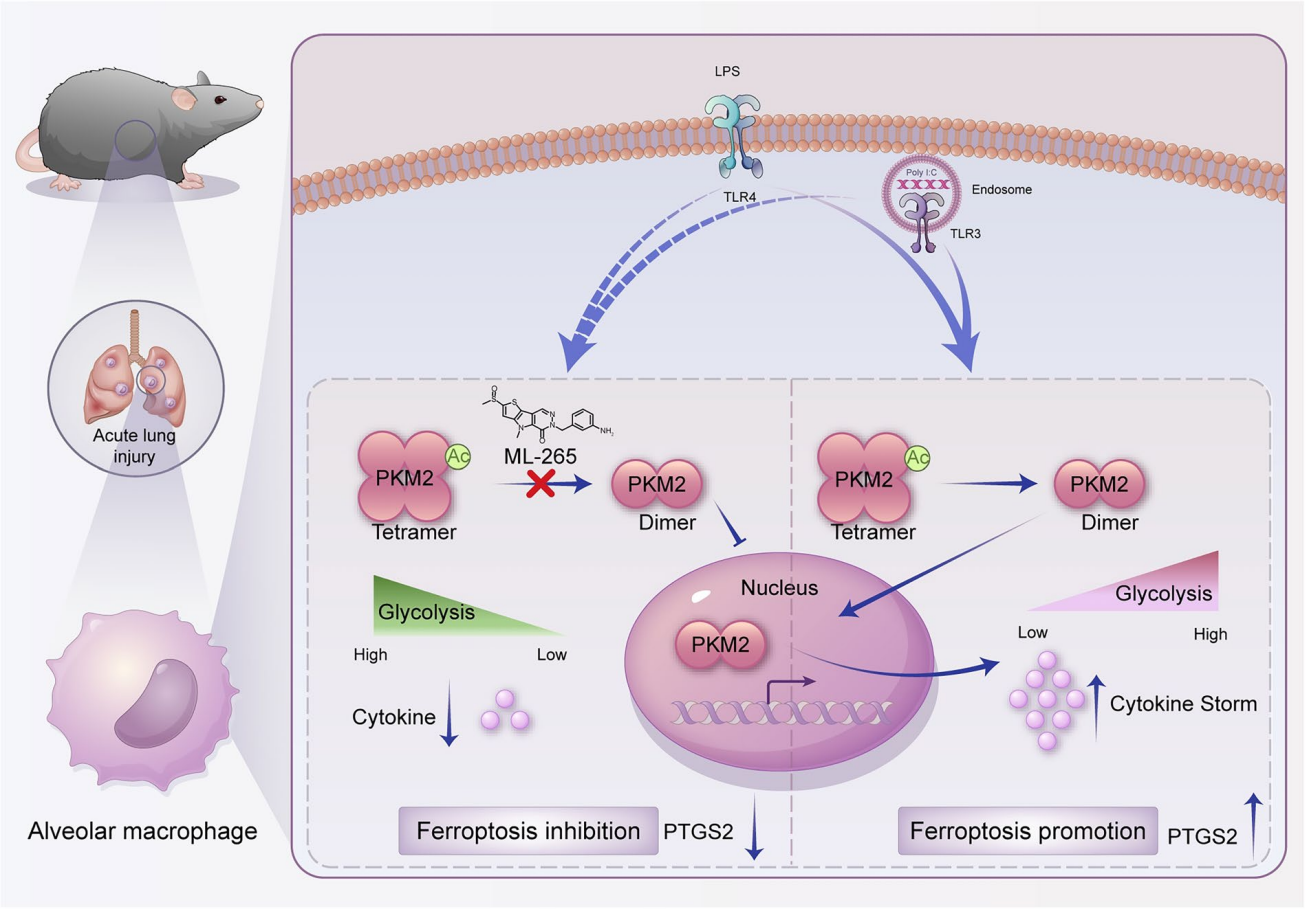
Schematic diagram of the study. Poly I:C (TLR3) and LPS (TLR4) stimulation increases glycolysis and leads to translocation of glycolytic enzyme PKM2 dimer into the nucleus of alveolar macrophage, which induces pro-inflammatory cytokine genes and ferroptosis genes expression. Induction of tetrameric PKM2 by ML-265 blocks its nuclear translocation and engagement of glycolysis, inhibiting ferroptosis and cytokine storm, and development of lung injury *in vivo*.

The COVID-19 pandemic revived research into CS. Ferroptosis may exacerbate the progression of the inflammatory response in macrophages [35–37]. Recently, macrophage metabolic reprogramming, particularly glycolysis, has emerged as a promising therapeutic strategy for combating ferroptosis during the CS [38, 39]. However, the correlation between CS associated with an ALI and ferroptosis remains unclear. In the current study, we demonstrated that ferroptosis inhibitors are particularly efficient at mitigating CS-associated ALI and improving survival. Importantly, we found that PKM2 translocates to the nucleus, enhancing the glycolytic metabolism of macrophages under different TLR stimulation conditions. Our study also demonstrated that treatment with ML-265 (an inhibitor of the tetrameric conformation of PKM2) markedly inhibited PKM2 nuclear entry and consequently suppressed ferroptosis (Fig. 7). In summary, this study provides novel insights into the non-canonical metabolic function of PKM2 in the modulation of ferroptosis and may aid in the development of drugs for the treatment of CS-associated lung injury.

Ferroptosis is a form of immunogenic cell death that can trigger inflammatory responses by releasing DAMPs [40], which may play an important role in hyperinflammation such as the CS. Accordingly, ferroptosis may be involved in multiple system diseases, e.g., acute kidney injury [41], ALI [42] and ischaemia–reperfusion injury (IRI) [11]. Consistent with previous studies, our findings confirmed ferroptosis activation in the mouse model by poly (I:C) and LPS challenge, mimicking severe infections in patients, via MDA and 4-NHE staining, and an immunofluorescence assay. Thus, agents that inhibit ferroptosis are promising new options for the treatment of the CS. Indeed, some strategies for inhibiting ferroptosis have shown potential as COVID-19 treatments. Two ferroptosis inhibitors, rosiglitazone (an ACSL4 inhibitor) and rosiglitazone, can reduce the viral yield of coronaviruses, including SARS-CoV-2 [43]. However, their use has raised major safety concerns, including increased risk of myocardial infarction, stroke, edema, congestive heart failure, reduced bone mineral density, increased fracture rate, and death [44], limiting their clinical application. Meanwhile, deferoxamine and deferiprone may alleviate hyper-ferritinemia by scavenging excess iron from the body in SARS-CoV-2 infection resulting in cytokine release syndrome [45, 46]. However, the clinical utility of iron chelation in ameliorating the CS has only been shown in small pilot studies, and evidence from large clinical trials remains lacking [47].

Elevated PTGS2 transcription is a hallmark of ferroptosis in malignant neoplasms [48], inflammatory [49], neurological diseases [50]. In addition, PTGS2 may mediate ferroptosis in neural cells after traumatic injury [51]. The chemical inhibition of system Xc- by erastin (a prototype trigger of ferroptosis) also results in PTGS2 upregulation in cancer cells [52]. Herein, we found that poly I:C and LPS insults resulted in leukopenia and thrombocytopenia; an upregulation of 4HNE, MDA, and PTGS2 mRNA levels; lung tissue injury; and pro-inflammatory factor abnormalities. Dang *et al.* recently reported that PKM2-activated macrophages drive abdominal aortic aneurysm progression by promoting vascular lipid peroxidation and ameliorating macrophage antioxidant capacity in vascular adventitia, as evidenced by increased gene expression of Ptgs2 and decreased gene expression of Gpx4 and Slc7a11 [53]. The mechanism may be related to PKM2, which facilitates the accumulation of iron and consequent ferroptosis. In contrast, Wang *et al*. found that tumor cells with downregulated PKM2 showed significant ferroptosis, which may account for the cellular metabolic pathway from glycolysis to OXPHOS, which has a profound impact on cellular ROS stress [22]. Overall, given the complexity of the mechanisms of ferroptosis and the specificity of PKM2 regulation in different cell types, whether other mechanisms exist through which PKM2 may induce ferroptosis-related genes expression requires further research.

A tightly controlled PKM2 conformation balance among PKM2 tetramers, dimers, and monomers is critical for PKM2 functions [54]. In an inflammatory context, mitochondrial ROS promote destabilization of the PKM2 tetramer, favoring its dimerization and subsequent nuclear translocation in macrophages [55]. PKM2 nuclear entry exerts different functions, such as histone phosphorylation [56] and regulation of gene transcription, in addition to encoding enzymes involved in glycolysis, including Glut1 and Ldha [57], also Il1b and Il12 [26, 58]. Moreover, previous studies in tumor cells have demonstrated that dimeric PKM2 complexed with b-catenin, STAT3, Oct4, NF-kB/p65, and HIF-1a to regulate the expression of target genes. However, other phenotypic mechanisms controlled by the nuclear function of PKM2 remain unexplored. In this study, we established a positive correlation between PKM2 nuclear entry and ferroptosis, which was reversed by treatment with a pharmacological dimeric PKM2 inhibitor. Notably, Leu and colleagues found that the treatment of cells with TEPP46, another pharmacological dimeric PKM2 inhibitor (akin to ML-265), reversed the increased ferroptotic cell death rate caused by glutamate [59]. Additionally, our RT-PCR assay demonstrated that the inhibition of dimeric PKM2 was significantly associated with decreased transcript expression of PTGS2. Whether the presence of dimeric PKM2 in the nucleus of macrophages facilitates transcription of ferroptosis sensitivity genes remains to be addressed.

## Conclusion

In conclusion, as a critical enzyme in glycolysis, dimeric PKM2 nuclear entry contributes to the expression of pro-inflammatory cytokines and ferroptosis activation. We established that the treatment of mice with ML-265 induced PKM2 to form a highly active tetrameric form, rebalancing anaerobic glycolysis and oxidative phosphorylation. PKM2 assists ferroptosis-related gene expression and may participate in ferroptosis activation during the CS. Notably, in PBMC from patients with a lung infection, PKM2 fluorescence in the nucleus was correlated with increased pro-inflammatory cytokine expression level. We propose that PKM2 is a key regulatory node that integrates metabolic reprogramming with intranuclear functions to regulate ferroptosis. Targeting PKM2 could be explored as a potential means of preventing or alleviating the hyper-inflammatory state or CS syndrome with aberrant ferroptotic cell death.

## Material and methods

### Animals

Eight-week-old male C57Bl/6 (WT) mice were purchased from Shanghai SLAC Laboratory Animal Co. Ltd. Isoflurane was used to anesthetize the animals to minimize their suffering. Animal experiments were performed in accordance with the Guide for the Care and Use of Laboratory Animals issued by the Ministry of Science and Technology of China. The study protocol was approved by the Institutional Animal Care and Use Committee of Shanghai Jiao Tong University School of Medicine (Permit Number: A-2019-041).

### Cytokine Storm Model

The CS model was induced by sequential challenge with poly I:C and LPS, as previously described [6]. Briefly, mice were intravenously injected with 10 mg/kg of high-molecular-weight poly I:C (InvivoGen). LPS derived from Escherichia coli 055:B5 (Sigma-Aldrich) diluted in phosphate-buffered saline (PBS) was injected intraperitoneally at 5 mg/kg 24 h after poly I:C injection. Mice were administered ferrostatin-1 (Fer-1, Selleck Chemicals) at 2.5 mg/kg by intraperitoneal injection [13], ML-265 (PKM2 monomer/dimer inhibitor, absin) at 50 mg/kg by intraperitoneal injection [60] or deferasirox (iron chelator, Deferasirox Dispersible Tablets, Novartis Pharma Schweiz) at 10 mg/kg by gavage [61]. The control group was treated with PBS. The plasma cytokines levels were measured using the Bio-Plex Pro Mouse Chemokine Panel 23-plex kit according to the manufacturer’s protocols (BIO-RAD).

### Cytokine and Metabolic Genes Expression

Single-cell suspensions were prepared from spleens and lungs using standard procedures [62]. After red blood cell lysis, cells were used for RNA extraction. Total RNA was isolated from the cells using TRIzol reagent (Invitrogen), and cDNA was synthesized using a high-capacity cDNA reverse transcription kit (Invitrogen). Quantitative real-time PCR was conducted using TB Green on the Roche LightCycler 480 system. The primers used for the indicated genes are listed in Supplemental Table 1.

### Histology, Immunohistochemistry, and Immunofluorescence

Fresh mouse lung tissue was fixed overnight in 2% paraformaldehyde. Tissue samples were embedded in paraffin and sectioned to slices 4-μm-thick, followed by hematoxylin and eosin staining. For immunohistochemistry, the primary antibodies, 4-HNE (Abcam, 1:200) or MDA (Adipogen, 1:100), were incubated overnight at 4 °C. Staining was performed using the Vectastain Elite ABC kit and DAB peroxidase substrate kit (Vector Laboratories). The images were acquired using a Leica DM750 microscope (Leica ICC50 W) camera.

For immunofluorescence, lung slices were dewaxed with xylene, graded with ethanol to water, and antigens were repaired with a buffer (pH 6.0). After being rinsed three times for 5 min in TBS, the sections were sealed with a blocking buffer (0.3% Triton X-100, 1% BSA, 1% FBS, and 0.1 M Tris HCL) at room temperature, incubated with the aqueous solution, and then incubated overnight with rabbit anti-4-HNE antibodies (Alpha Diagnostic International,1:200 dilution) or anti-PKM2 antibodies (CST,1:200 dilution) at 4 °C. After three washes with PBS, the cells were incubated at room temperature with fluorescently labeled secondary antibodies for 1 h. Nuclei were subsequently stained with 6-diamino-2-phenylindole in the dark. For mitochondrial ROS detection, lung tissues were mixed with MitoSox at 100 nM (Invitrogen) for 15 min at 37 °C and then fixed in 2% paraformaldehyde for 15 min at 37 °C. The images were captured using a fluorescence microscope.

### Transmission Electron Microscopy

Fresh myocardium samples (1 mm × 1 mm × 1 mm) were quickly and carefully collected and placed in tubes containing a fixing solution for 4 h. Then, the lung tissue samples were permeabilized, dehydrated, and embedded overnight. The samples were viewed using a Tecnai 10 Electron Microscope (FEI, Hillsboro, OR, USA) at the Electron Microscopy Core Facility, Shanghai Jiao Tong University School of Medicine.

### Flow Cytometry Sorting and Analysis

For AM sorting from the lung cells of the mouse model, the cells were first stained with an Fc blocker in MACS buffer (1 × PBS with 0.5% BSA, 2 mM EDTA). Then the cells were resuspended in 50µL MACS buffer with diluted indicated flow cytometry antibodies and incubated for 30 min at 4 °C. AM were gated as CD45^+^(APC Anti-Mouse CD45 antibody, clone 30-F11, Abcam) Ly6G^−^ (PE-Cy7 Anti-Mouse Ly6G antibody, clone 1A8, Biolegend) CD11b^lo−int^ (FITC Anti-Mouse/Human CD11b Antibody, clone M1/70, Biolegend) Siglec F^+^ (PE Anti-Mouse Siglec-F, clone E50-2440, BD Pharmingen) live cells. Cells were washed twice with MACS buffer and sorted using a Beckman Coulter MoFlo Astrios EQ.

### Analysis of Extracellular Acidification rate (ECAR) and Oxygen Consumption rate (OCR) with the Seahorse XF Platform

Cellular and mitochondrial bioenergetics were measured using a Seahorse XF96 extracellular flux analyzer. Glycolysis and mitochondrial stress tests were performed according to manufacturer’s instructions. For glycolysis stress test, cells were incubated in glucose-free Seahorse assay media supplemented with 1 mM pyruvate at 37 °C in an incubator without CO_2_ for 1 h prior to the assay. Injectors were loaded to add 20 mM glucose, 1 μM oligomycin and 100 mM 2 deoxy-glucose (2-DG). For the mitochondrial stress test, injectors were loaded with 20 mM glucose, 1 µM oligomycin, 1 µM FCCP, 1 µM rotenone and 2 µM antimycin A during mitochondrial stress test. The baseline ECAR and OCR values were averaged between technical replicates for the first three successive time intervals.

### Subcellular Fractionation

Subcellular fractionation was carried out to isolate nuclear-enriched lysates as previously described. First, a cell lysis buffer suspension was used to extract the soluble cytoplasmic protein fraction. After incubation on ice for 10 min, samples were centrifuged. The supernatant containing the soluble cytoplasmic fraction was removed. The residual nuclei pellet was treated according to the manufacturer's instructions using Turbo DNAse. Eventually, the complete solubilization of the nuclei was achieved by introducing nuclear lysis buffer, followed by its incubation at 4 °C for 10 min. The final step involved mechanical centrifugation of the samples and the subsequent collection of the supernatant comprising the nuclear lysate.

### Western Blot Analysis

Western blot analysis was performed on cells that were first lysed in RIPA buffer. The analysis was performed according to a previously described methodology. The samples were electrophoresed on either 7.5% or 10% gels and transferred onto PVDF membranes. The membranes were then blocked at room temperature with a 5% milk solution in TBS-Tween for a duration of 1 h prior to the commencement of primary antibody incubation, which was performed at 4 °C overnight. An HRP-conjugated secondary antibody was added and subsequently incubated at room temperature for 1 h. Final visualization of the western blots was achieved using chemiluminescence reagents.

### Clinical Cohort

We conducted a cohort study that included 14 patients diagnosed with a lung infection and three healthy controls at the Department of Emergency and Critical Care Medicine, Renji Hospital from April to September 2023. Nine patients presented distinct characteristics indicative of CS, as per the defined predictive criteria [34]. Lung infection patients enrolled in this study were all initial infections during hospitalization. Participants under the age of 18 years, pregnant women, with recurrent infections and those with a documented terminal illness, a history of malignant tumors, human immunodeficiency virus, autoimmune diseases, and diseases of the blood were excluded. During hospitalization, we recorded data on hospital admission, including demographic characteristics (sex and age), laboratory test results, pathogen details and treatment regimens. This study was approved by the Ethics Committee of Renji Hospital (KY2023-053-B).

### Human Peripheral Blood Cells Isolation and Analysis

Five-milliliter whole blood samples were obtained from patients on the day of admission and healthy volunteers using ACD tubes (BD Vacutainer) and fractionated over Ficoll gradients to obtain peripheral blood mononuclear cells (PBMCs).

CD14^+^ cells were isolated from PBMCs using a positive selection kit (EasySep™ Human CD14 Positive Selection Kit II; STEMCELL). PBMCs or CD14^+^ cells (1 × 10^4^ cells-5 × 10^4^ cells) were centrifuged onto glass slides using a Cytospin 4 Cytocentrifuge (Thermo Scientific) and subsequently fixed for immunofluorescence staining targeting PKM2. Subsequently, cells were distinguished as neutrophils (Ly6G^+^), B cells (CD19^+^), or T cells (CD3^+^). The cell nuclei were stained with DAPI.

CD14^+^ cells were also prepared for flowcytometry analysis. Briefly, the cells were permeabilized for 1 h using the FOXP3/transcription factor staining buffer set (Thermo Fisher Scientific) and then stained for 1 h with antibodies against PKM2 (CST,1:200 dilution). The cells were washed twice with a MACS buffer and analyzed by flow cytometry. Data were acquired using a BD Fortessa X20 (BD Biosciences) and analyzed using the FlowJo software (Tree Star, Inc.).

### Library Preparation and RNA-Seq Analysis

In this study, three patients exhibiting CS and age-matched healthy controls were selected. CD14^+^ cells were isolated from the individuals' peripheral blood samples. In addition, three mice each from the IC-LPS model group and the PBS control group were used in this study. Alveolar macrophages (AM) were extracted from these mice as previously outlined. RNA was then extracted from these samples, and 1 μg of total RNA was assessed for quality (RIN > 7) using an Agilent Bioanalyser 2100 system. This RNA sample was then used to build barcoded sequencing libraries following the instructions of Illumina's TruSeq Stranded mRNA Library Prep Kit.

Raw RNA-seq data were analyzed using common RNA-seq analysis tools. The quality of the FastQ files was determined using FastQC version 0.11, which revealed Phred quality scores above 20 points, indicating a base call accuracy of 99%. Low-quality reads were trimmed using Trimmomatic, version 0.36. The RNA-seq files were aligned to either the human or mouse reference genome (version hg38) using HISAT2 (version 2.2.1.0). Gene read counts were performed using HTSeq version 0.9.1. Differentially expressed genes were analyzed using R software with the DESeq2 package in Bioconductor. Subsequently, GSEA was performed using GSEA (version 3.0; Broad). The gene expression data were normalized with a log2 fold change (FC > 1) along with an adjusted P-value (false discovery rate of ≤ 0.05), with the annotation information also provided.

### Statistical Analysis

Results are expressed as the mean±standard deviation (SD). Differences between groups were evaluated by Student’s t-test or one-way analysis of variance using GraphPad Prism 4 software. The log-rank Mantel-Cox test was used to compare Kaplan-Meier curves. In clinical cohort, categorical variables were described as frequencies, and differences were assessed using the chi-square test. Continuous variables are presented as medians with interquartile ranges, and differences were assessed using Wilcoxon rank-sum tests. The correlation between intracellular PKM2 expression and inflammatory biomarker levels was determined using Spearman's rank correlation. P values less than 0.05 were considered significant.

## Supplementary Information

Below is the link to the electronic supplementary material.

Supplementary file1 (DOCX 9900 KB)

Supplementary file2 (DOCX 24 KB)

## Data Availability

All data generated or analyzed during this study are included in this published article.
