# Supplementary material for: Pyruvate Kinase M2 Nuclear Translocation Regulate Ferroptosis-Associated Acute Lung Injury in Cytokine Storm
Source: Inflammation. 2024 Mar 14;47(5):1667–84. doi: 10.1007/s10753-024-02000-x (PMC11549213; doi:10.1007/s10753-024-02000-x)
Supplement: Supplementary file 1 — Supplementary file1 (DOCX 9900 KB) [file 10753_2024_2000_MOESM1_ESM.docx]

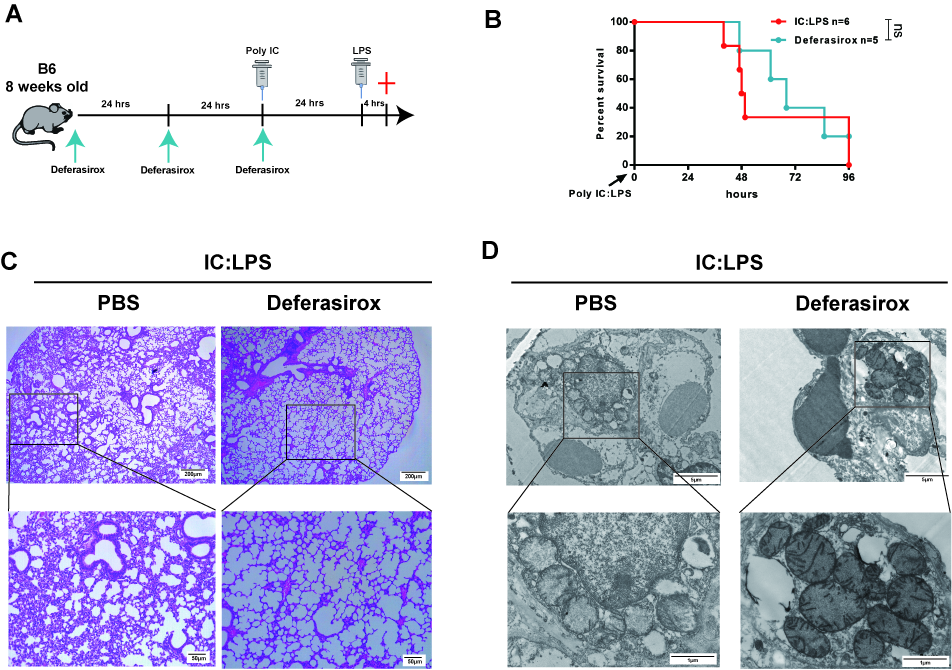


**Sup Fig.1 Deferasirox treatment attenuated IC:LPS‑induced lung injury in mice**

(A) Experimental protocol. LPS(5mg/kg) was injected intraperitoneally at 24 h after poly I:C (10 mg/kg) injection. Where indicated, the mice were administered a daily gavage of deferasirox (10mg/kg) for three consecutive days, starting two days before poly I:C treatment. Mice were scarified 4 h after the second challenge. (B) Survival of animals challenged with IC:LPS between two groups. H&E staining(C) and mitochondrial morphology(D) in lung sections were measured.


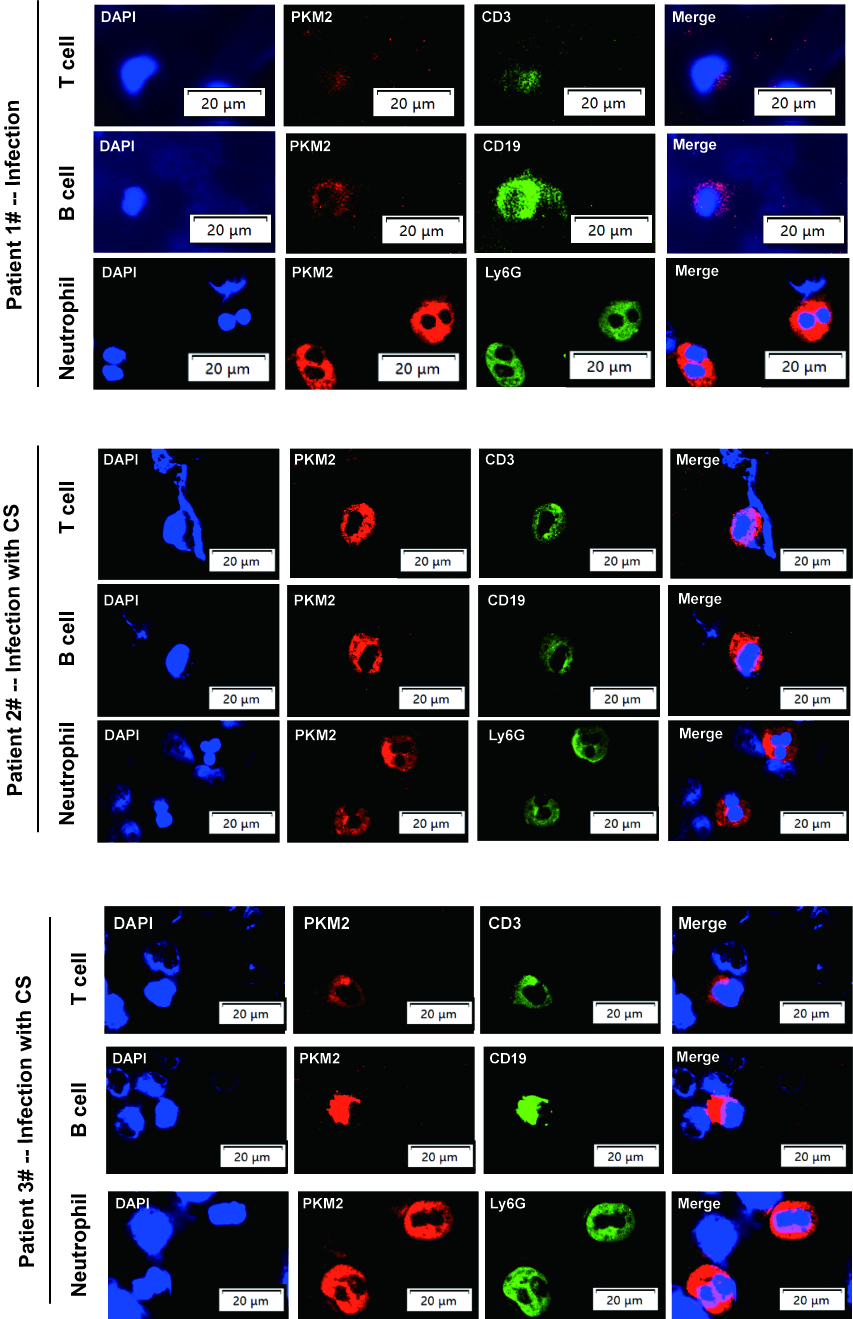


**Sup Fig.2 Confocal imaging of PKM2 (red) and DAPI (blue) labeling in neutrophils (green, Ly6G^+^), B cells (green, CD19^+^), or T cells (green, CD3^+^).**
