# Supplementary material for: Pyruvate Kinase M2 Nuclear Translocation Regulate Ferroptosis-Associated Acute Lung Injury in Cytokine Storm
Source: Inflammation. 2024 Mar 14;47(5):1667–84. doi: 10.1007/s10753-024-02000-x (PMC11549213; doi:10.1007/s10753-024-02000-x)
Supplement: Supplementary file 2 — Supplementary file2 (DOCX 24 KB) [file 10753_2024_2000_MOESM2_ESM.docx]

**Table S1. Primers used for qRT-PCR.**

| ***Glut1*** | Forward: 5’- CAGTTCGGCTATAACACTGGTG |
| --- | --- |
|  | Reverse: 5’- GCCCCCGACAGAGAAGATG |
| ***Hk1*** | Forward: 5’- TTCGAGAAGATGGTGAGCGG |
|  | Reverse: 5’- GGAGAGTTCCCATCCCGTTT |
| ***Hk2*** | Forward: 5’- TGATCGCTGCTTATTCACGG |
|  | Reverse: 5’- AACCGCCTAGAA ATCTCCAGA |
| ***Pkm2*** | Forward: 5’- GTGGCTCGGCTGAATTTCTCT |
|  | Reverse: 5’- CACCGCAACAGGACGGTAG |
| ***Meis1*** | Forward: 5’- CAGAAAAAGCAGTTGGCACA |
|  | Reverse: 5’- TGCTGACCGTCCATTACAAA |
| ***Pfkfb3*** | Forward: 5’- TCTAGAGGAGGTGAGATCAG |
|  | Reverse: 5’- CCTGCCACTCTTATCTTCTG |
| ***Pgk*** | Forward: 5’- CTGTGGTACTGAGAGCAGCAAGA |
|  | Reverse: 5’- CAGGACCATTCCAAACAATCTG |
| ***Hif1α*** | Forward: 5’- CTACTGCAGGGTGAAGAATTACTCAGAGC |
|  | Reverse: 5’- GTGCAATTGTGGCTACCATGTACTGCTG |
| ***Pdk1*** | Forward: 5’- GGACTTCGGGTCAGTGAATGC |
|  | Reverse: 5’- TCCTGAGAAGATTGTCGGGGA |
| ***Tfam*** | Forward: 5’- AACACCCAGATGCAAAACTTTCA |
|  | Reverse: 5’- GACTTGGAGTTAGCTGCTCTTT |
| ***Gpx1*** | Forward: 5’- CCACCGTGTATGCCTTCTCC |
|  | Reverse: 5’- AGAGAGACGCGACATTCTCAAT |
| ***Sco1*** | Forward: 5’- GGCACAGCCAGTGCATTCCTGCCTGC |
|  | Reverse: 5’- GCATCACACTCGTCAATATCCTC |
| ***Atp5d*** | Forward: 5’- CTCCTCTGTGCAGTTACTAGCTGAA |
|  | Reverse: 5’- ACTGCGCCTTCTCCAGGTT |
| ***Cox-2*** | Forward: 5’- ATAACCGAGTCGTTCTGCCAAT |
|  | Reverse: 5’- TTTCAGAGCATTGGCCATAGAA |
| ***G6pd*** | Forward: 5’- CACAGTGGACGACATCCGAAA |
|  | Reverse: 5’- AGCTACATAGGAATTACGGGCAA |
| ***G6pdx*** | Forward: 5’- CACAGTGGACGACATCCG AAA |
|  | Reverse: 5’- AGCTACATAGGA ATTACGGGCAA |
| ***Pgd*** | Forward: 5’-CGTAAGGCCCTCTATGCTTC |
|  | Reverse: 5’- TGAAGTTCTGGGTTTCGCTC |
| ***Cpt1-a*** | Forward: 5’- CCAGGCTACAGTGGGACATT |
|  | Reverse: 5’- GAACTTGCCCATGTCCTTGT |
| ***Cpt1-b*** | Forward: 5’- CGTTCCTGTACCAACGAGTC |
|  | Reverse: 5’- CAGAAAGTACCTCAGCCAGG |
| ***Cpt2*** | Forward: 5’- ACCATGCACTACCAGGACAG |
|  | Reverse: 5’-TATCAAACCAGGGGCCTGAGA |
| ***Ldha*** | Forward: 5’-GTGCCCAGTTCTGGGTTAAG |
|  | Reverse: 5’-CTGGGTCCTGGGAGAACAT |
| ***Odc*** | Forward: 5’- GACGAGTTTGACTGCCACATC |
|  | Reverse: 5’- CGCAACATAGAACGCATCCTT |
| ***Gapdh*** | Forward: 5’-TGTGTCCGTCGTGGATCTGA |
|  | Reverse: 5’- CCTGCTTCACCACCTTCTTG |
| ***IL10*** | Forward: 5- CTTACTGACTGGCATGAGGATCA-3’ |
|  | Reverse: 5’- GCAGCTCTAGGAGCATGTGG-3’ |
| ***IL6*** | Forward: 5’- CTGCAAGAGACTTCCATCCAG-3’ |
|  | Reverse: 5’- AGTGGTATAGACAGGTCTGTTGG-3 |
| ***Il1a*** | Forward: 5’- TCTATGATGCAAGCTATGGCTCA-3’ |
|  | Reverse: 5’- CGGCTCTCCTTGAAGGTGA-3’ |
| ***TNFa*** | Forward: 5’- CAGGCGGTGCCTATGTCTC-3’ |
|  | Reverse: 5’- CGATCACCCCGAAGTTCAGTAG-3’ |
| ***IL18*** | Forward: 5’- GTGAACCCCAGACCAGACTG-3’ |
|  | Reverse: 5’- CCTGGAACACGTTTCTGAAAGA-3’ |
| ***GM-CSF*** | Forward: 5’-TGTGTCCGTCGTGGATCTGA-3’ |
|  | Reverse: 5’- CCTGCTTCACCACCTTCTTG-3’ |
| ***IFNr*** | Forward: 5’-GCCACGGCACAGTCATTGA-3’ |
|  | Reverse: 5’-TGCTGATGGCCTGATTGTCTT-3’ |
| ***Ptgs2*** | Forward: 5’- GGAGAGAAAGAAATGGCTGC -3’ |
|  | Reverse: 5’- ATCTAGTCTGGAGTGGGAGG -3’ |
